# A quantitative structure- property relationship of gas chromatographic/mass spectrometric retention data of 85 volatile organic compounds as air pollutant materials by multivariate methods

**DOI:** 10.1186/1752-153X-6-S2-S4

**Published:** 2012-05-02

**Authors:** Maryam Sarkhosh, Jahan B Ghasemi, Mahnaz Ayati

**Affiliations:** 1 Chemistry Department, Faculty of Sciences, K.N.Toosi University of Technology, Tehran, Iran

## Abstract

A quantitative structure-property relationship (QSPR) study is suggested for the prediction of retention times of volatile organic compounds. Various kinds of molecular descriptors were calculated to represent the molecular structure of compounds. Modeling of retention times of these compounds as a function of the theoretically derived descriptors was established by multiple linear regression (MLR) and artificial neural network (ANN). The stepwise regression was used for the selection of the variables which gives the best-fitted models. After variable selection ANN, MLR methods were used with leave-one-out cross validation for building the regression models. The prediction results are in very good agreement with the experimental values. MLR as the linear regression method shows good ability in the prediction of the retention times of the prediction set. This provided a new and effective method for predicting the chromatography retention index for the volatile organic compounds.

## Introduction

Volatile organic compounds (VOCs) are molecules that have a high vapor pressure and low water solubility, like organic chemicals in general. There are many different compounds which may be classified as volatile organic compounds (VOCs). The compounds the nose detects as smells are generally VOCs. Many VOCs are human-made chemicals that are used and produced in the manufacture of paints, pharmaceuticals, and refrigerants. VOCs typically are industrial solvents, such as trichloroethylene; fuel oxygenates, such as methyl tert-butyl ether (MTBE), or by-products produced by chlorination in water treatment, such as chloroform.

VOCs are common ground-water contaminants. Many volatile organic compounds are also hazardous air pollutants. VOCs also play a major role in the formation of various secondary pollutants through photochemical reactions in the presence of sunlight and nitrogen oxides. Furthermore, some VOCs could contribute to the atmospheric ozone depletion and the build-up persistent pollutions in remote areas. Therefore, these compounds have been an important environmental issue over the last two decades and have attracted significant attention from different research groups. Although scientist are provided a sensitive and specific analytical method for identifying and measuring the VOCs [1-5], but there are some limitations for these methods. For example, sorbents are normally selective in, although not limited to, adsorbing/absorbing certain classes of VOCs or sometimes high sample volumes of air are needed for identification or measuring the VOCs. Besides the above mentioned method, the experimental determination of retention time is time-consuming and expensive. Alternatively, quantitative structure–retention relationship (QSRR) provides a promising method for the estimation of retention time based on descriptors derived solely from the molecular structure to fit experimental data [6-8]. A QSRR study involves the prediction of chromatographic retention parameters using molecular structure. QSRR studies are widely investigated in gas chromatography (GC) [9-11] and high-performance liquid chromatography (HPLC) [12]. The chromatographic parameters are expected to be proportional to a free energy change that is related to the solute distribution on the column.

Model development in QSAR/QSPR studies comprises different critical steps as (1) descriptor generation, (2) data splitting to calibration (or training) and prediction (or validation) sets, (3) variable selection, (4) finding appropriate model between selected variables and activity/property and (5) model validation.

In the present work, a QSRR study has been carried out on the GC/MS system retention times (*t*_R’_s) for 85 volatile organic compounds by using structural molecular descriptors. We applied a linear MLR and a nonlinear algorithms ANN along with stepwise regression as variable selection method.

## Computer hardware and software

All calculations were run on a Toshiba personal computer with a Pentium IV as CPU and windows XP as operating system. The molecular structures of data set were sketched using ChemDraw (Ver. 11, supplied by Cambridge Software Company). The sketched structures were exported to Chem3D module in order to create their 3D structures. Energy minimization was performed using MM+ molecular mechanics and AM1 semi-empirical to obtain the root mean square (RMS) gradient below 0.01 kcal/mol Å. The next step in developing a model is generation of the corresponding numerical descriptors of the molecular structures. Molecular descriptors define the molecular structure and physicochemical properties of molecules by a single number. A wide variety of descriptors have been reported for using in QSAR/QSPR analysis. Here, 477 descriptors were generated for each compound, using Dragon (ver. 3) software (Milano Chemometrics and QSAR research group, http://www.disat.unimib.it/chm/). SPSS (ver.16, http://www.spss.com) other calculations were performed in PLS_Toolbox (ver. 4.1, Eigenvector Company) and MATLAB (version 7.8, Math Works, Inc.) environment. SPSS (ver.16, http://www.spss.com) was used in variable selection step to select the most relevant descriptors. Other calculations were performed in PLS_Toolbox (ver. 4.1, Eigenvector Company) and MATLAB (version 7.8, Math Works, Inc.) environment.

## Data set

The retention times of volatile organic compounds have been obtained from reference [13], we have also added the 8 compounds (1, 4-difluorobenzene, chlorobenzene,1,4-dichlorobenzene,4-bromofluorobenzene, 1,2-dichlorobenzene, dichloroethane, toluene, fluorobenzene ) that are generally used as internal standards in the GC/MS analysis. All 85 VOCs are listed in Table 1. The analytes have been extracted from the matrices and components are separated, identified and measured by a wide bore capillary column of a gas chromatography/mass spectrometer detector system.

**Table 1:** The data set and the corresponding experimental and predicted t_R_ values by MLR and ANN methods.

NO.	name	t_R_ (min) Exp.	t_R_ MLR	t_R_ ANN	NO.	name	t_R_ (min) Exp.	t_R_ MLR	t_R_ ANN
m1	Dichlorodifluoromethane	1.35	1.50	1.52	m44	p-Xylene	23.54	22.69	22.8
m2	Carbon disulfide	4.11	2.71	2.67	m45	m-Xylene	23.54	22.69	22.6
m3	Allyl chloride	4.11	6.57	6.57	m46	o-Xylene^a^	25.16	22.66	22.5
m4	Methylene chloride	4.40	3.93	4.12	m47	Styrene	25.3	23.09	22.9
m5	1,1-Dichloroethene	4.57	6.75	6.79	m48	Bromoform^b^	26.23	26.19	26.2
m6	Acetone	4.57	3.66	4.22	m49	Isopropylbenzene (Cumene)	26.37	27.00	26.76
m7	trans-1,2-Dichloroethene	4.57	8.77	8.76	m50	cis-1,4-Dichloro-2-butene	27.12	27.18	27.39
m8	Acrylonitrile	5.00	4.52	4.80	m51	Trichlorofluoromethane^a^	2.42	6.04	5.75
m9	1,1-Dichloroethane	6.14	8.62	8.16	m52	1,1,2,2-Tetrachloroethane	27.29	25.7	25.79
m10	Vinyl acetate	6.43	11.47	11.50	m53	Bromobenzene	27.46	26.85	26.92
m11	2,2-Dichloropropane	8.10	12.42	12.61	m54	1,2,3-Trichloropropane^a^	27.55	27.46	26.61
m12	Chloromethane	1.49	-0.34	-0.12	m55	n-Propylbenzene	27.58	27.92	27.64
m13	cis-1,2-Dichloroethene^a^	8.25	9.82	9.86	m56	2-Chlorotoluene	28.19	27.88	27.96
m14	Propionitrile	8.51	5.61	5.99	m57	trans-1,4-Dichloro-2-butene	28.26	26.64	26.75
m15	Chloroform	9.01	7.78	7.50	m58	1,3,5-Trimethylbenzene	28.31	26.62	28.48
m16	Methacrylonitrile	9.19	8.47	8.74	m59	4-Chlorotoluene	28.33	27.85	27.94
m17	1,1,1-Trichloroethane^a^	10.18	13.58	12.60	m60	Pentachloroethane	29.41	28.41	28.17
m18	Carbon tetrachloride	11.02	11.75	11.23	m61	1,2,4-Trimethylbenzene^a^	29.47	26.75	26.61
m19	1,1-Dichloropropene	11.50	14.4	14.29	m62	Acrolein	3.19	3.33	3.44
m20	Benzene^a^	1.56	1.70	1.84	m63	sec-Butylbenzene	30.25	31.85	31.32
m21	Vinyl Chloride	12.09	11.39	11.53	m64	tert-Butylbenzene	30.59	30.14	29.86
m22	1,2-Dichloroethane	14.03	13.84	13.67	m65	p-Isopropyltoluene^a^	30.59	31.10	30.85
m23	Trichloroethene	14.51	15.14	15.43	m66	1,3-Dichlorobenzene	30.56	31.78	31.94
m24	1,2-Dichloropropane	15.39	16.45	16.45	m67	1,4-Dichlorobenzene	31.22	32.65	32.88
m25	Dibromomethane	15.43	12.66	12.21	m68	Benzyl chloride	32.00	28.35	28.52
m26	Methyl methacrylate^a^	15.50	15.79	15.69	m69	n-Butylbenzene	32.23	32.68	32.35
m27	1,4-Dioxane	16.17	15.46	15.43	m70	1,2-Dichlorobenzene	32.31	32.00	32.17
m28	2-Chloroethyl vinyl ether	17.32	15.02	15.92	m71	1,2-Dibromo-3-chloropropane^a^	35.30	33.29	33.39
m29	4-Methyl-2-pentanone	17.47	16.13	16.81	m72	1,2,4-Trichlorobenzene	38.19	40.76	4102
m30	trans-1,3-Dichloropropene	2.19	2.08	1.94	m73	Iodomethane	3.56	6.13	5.83
m31	Bromomethane	18.29	18.63	18.53	m74	Hexachlorobutadiene^b^	38.57	-	-
m32	Toluene	19.38	15.38	15.90	m75	Naphthalene^a^	39.05	33.39	33.04
m33	cis-1,3-Dichloropropene	19.59	19.34	19.50	m76	1,2,3-Trichlorobenzene	40.01	23.49	40.8
m34	Ethyl methacrylate^a^	20.01	24.52	24.70	m77	1,4-Difluorobenzene^a^	13.26	13.68	13.6
m35	2-Hexanone	20.30	21.03	21.24	m78	Chlorobenzene-d5	23.10	23.49	23.28
m36	Tetrachloroethene	20.26	19.88	19.62	m79	1,4-Dichlorobenzene-d4	31.16	32.65	32.88
m37	1,3-Dichloropropane^a^	20.51	16.58	16.67	m80	4-Bromofluorobenzene	27.83	25.93	26.08
m38	Dibromochloromethane	21.19	21.94	21.62	m81	1,2-Dichlorobenzene-d4^a^	32.30	32.00	32.11
m39	1,2-Dibromoethane	21.52	20.89	20.21	m82	Dichloroethane-d4	12.08	8.62	8.76
m40	Chloroethane	2.21	2.83	3.03	m83	Acetonitrile^a^	4.11	3.23	4.00
m41	Chlorobenzene	23.17	23.52	23.61	m84	Toluene-d8	18.27	18.63	18.53
m42	1,1,1,2-Tetrachloroethane	23.36	22.88	22.81	m85	Fluorobenzene	13.00	14.04	14.11
m43	Ethylbenzene	23.38	23.26	23.06					

^a^ indicates prediction set

^b^ outlier

## Results and discussion

### Principal component analysis

**Figure 1:**
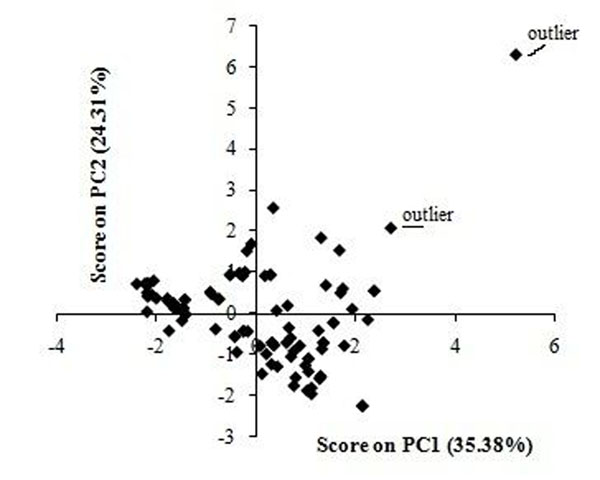
Scatter plot of VOCs on principal components^,^ plane

Principal components analysis (PCA) was performed on the calculated structural descriptors to the whole data set of VOCs (Table 1), for investigation the distribution in the chemical space, which shows the spatial location of samples to assist the separation of the data into training and test sets [14]. The PCA results show that two principal components (PC1 and PC2) describe 59.7% of the overall variances: PC1 =35.38%, PC2= 24.31% (Figure 1). Since almost all variables can be accounted for the first two PCs, their score plot is a reliable representation of the spatial distribution of the points for the data set. The plot of PC2 against PC1, Figure1, displays the distribution of compounds over the first two principal components space. According to the results of PCA, 2 molecules were removed as outliers(Figure 1) the remaining molecules were divided into a 67 compounds as training set to develop the models and 16 compounds as prediction set to evaluate the models.

## Descriptor selection

477 descriptors were calculated by Dragon for each molecule, therefore we have 85×447 data matrix **X.** The rows and columns of this matrix are the number of molecules and molecular descriptors respectively. All descriptors have been checked to ensure: (a) that values of each descriptor are available for each structure (the investigation of missing values) and (b) that there is a significant variation in these values over studied molecules. Descriptors which has zero value for each structure or shows constant values over molecules in the under study data set are discarded. **X** and **y**(dependent variable) were preprocessed by autoscaling. We used stepwise regression [15] for variable selection. PCA [16] is applied for reduction of the descriptor space (variable space).

In the variable selection step, feature selection (FS) [17-19] and feature extraction (FE) are commonly used methods to handle a large number of calculated descriptors in QSAR/QSPR studies. In FE methods, by the use of principal component analysis (PCA), the information contained in the descriptors data matrix is extracted into new orthogonal variables with lower dimensions.

**Figure 2:**
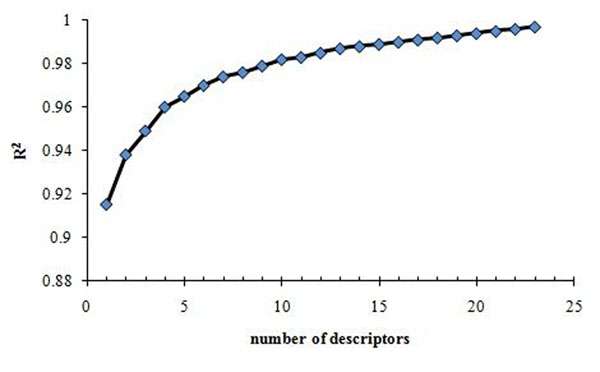
R^2^ as a function of the number of descriptors that were selected with stepwise regression.

In stepwise procedure a variable that entered the model in the earlier stages of selection may be deleted at the later stages. The calculations made for inclusion and elimination of variables are the same as forward selection and backward procedures. That is, the stepwise methods are essentially a forward selection procedure, but at each stage the possibility of deleting a variable, as in backward elimination, are considered. Stepwise addition of further descriptors is continued to find the best multi-parameter regression models with the optimum values of statistical criteria (highest values of R^2^ and the cross-validated R^2^_cv_). With stepwise regression 23 descriptors were selected and a simple ‘‘break point’’ technique was used to control the model expansion in the improvement of the statistical quality of the model, by analyzing the plot of the number of descriptors involved in the obtained models versus squared correlation coefficient values corresponding to those models (Figure 2). The model corresponding to the break point is considered the best optimum model, in this way 6 descriptors that have high contribution in the variance of dependent variable (t_R_) were selected and used to build the models.

## Multivariate regression models

Multiple linear regression (MLR), is the most frequently used modeling method in QSAR/QSPRs. MLR yields models that are simpler and easier to interpret than factor based methods like PCR(principal component regression) [20,21] and PLS(partial least squares) [22-27]. However, due to the co- linearity between structural descriptors, MLR is not able to extract useful information from structural data, and over fitting problem is encountered. ANN model used to handle the probable nonlinear relationship between descriptors and retention times. One of the most striking point of ANN is that its free from collinearity problem.

We have tested the stability of models by random selection of train and test sets to assure the resulting models are not just chance correlations. 100 models by random selection are constructed and the average of R^2^ and RMSEC are 0.971 and 0.162, respectively. The results show that the different selected test sets from the data have very similar R^2^ values. This indicates that the final model is reliable and has a satisfactory stability.

## Multiple linear Regression (MLR)

Multiple linear regression [28] are the most widely used and known modeling methods.

MLR is performed either to study the relationship between the response variable and predictor variables or to predict the response variable based on the predictor variables. The validation of the model was performed by cross-validation method. With cross-validation, one sample was kept out (leave-one-out) of the calibration and used for prediction. The process was repeated so that each of the samples was kept out once. The predicted values of left-out samples were then compared to the observed values using prediction error sum of squares(PRESS) which indicating the residuals are computed in cross-validation. PRESS eq. (1) and RMSE, root mean squared error, eq. (2) are defined as:(1)(2)

where ŷ_i_ is the estimated value of the *ith* object , *y_i_* is the corresponding reference value of this object and N is the number of the objects.

**Figure 3:**
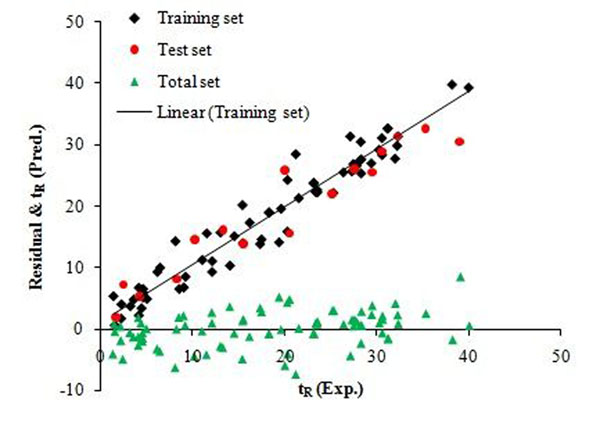
Experimental t_R_ values and residual vs. Predicted t_R_ values by MLR modeling

Table 2 shows the statistical parameters of the model corresponding to the 6 independent variables. The unstandardized coefficients and standard error that allows the comparison of the relative weight of the variables in the model are presented in Table 3. Plots of experimental *t*_R_ values and residuals (experimental *t*_R_–predicted *t*_R_) versus predicted *t*_R_ values, obtained by the MLR modeling, are shown in Figure 3 The agreement observed between the predicted experimental values in and the random distribution of residuals about zero mean confirms the good predictive ability of MLR modeling.

**Table 2:** The statistical parameters of MLR model

Variable selection method	Calibration method	R^2^ Calibration	R^2^ Prediction	R^2^ L-10-Out	R^2^ L-5-Out	R^2^ LOO	RMSEC^*^	RMSEP^**^	No. DS_s_
stepwise	MLR	0.972	0.957	0.962	0.964	0.964	0.16	0.247	6

*root mean squared error of prediction, **root mean squared error of prediction

**Table 3:** best MLR model for the prediction of VOCs retention times

Notation	Descriptors	Unstandardized Coefficients	Std. Error	mean effect
X1sol	salvation connectivity index	0.823	0.032	0.044
X5A	average connectivity index	0.146	0.029	0.004
Mor10m	3D MoRSE- signal 10/weighted by atomic masses	-0.079	0.026	-0.003
Mor21m	3D MoRSE- signal 21/weighted by atomic masses	-0.165	0.031	0.008
ATS2m	broto-moreau autocorrelation of a topological structure- log2/weighted by atomic masses	0.188	0.034	-0.015
MATS2m	Moran autocorrelation− lag 2/weighted by atomic masses	0.135	0.026	-0.009
	Constant	0.002	0.021	

To show the absence of correlation by chance between independent and dependents variables randomization test was performed. The MLR modeling was performed on the randomized data, the elements of the response vector were randomly mixed and the modeling was performed on the matrix **X** and new response vector [29]. The squared correlation coefficient of the model with the randomly selected data (R^2^_CR_) was much lower than those of the original model, it could be considered that the model was reliable, and had not been obtained by chance. The low mean of correlation value (R^2^_CR_=0.091) obtained by chance confirms this result.

**Figure 4:**
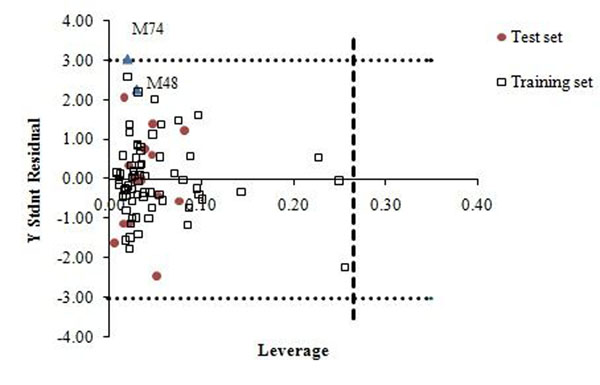
Plot of standardized residuals versus leverages. Dotted lines represent ±3 standardized residual, dash line represents warning leverage (h* ≈ 0.265).

One of the most important problems in QSAR/QSPR analysis is establishing the domain of applicability for each model [30]. In the absence of the applicability domain restriction, each model can formally predict the property of any compound, even with a completely different structure from those included in the training set. The analysis of the chemical applicability domain (AD) of the obtained model and the reliability of the predictions are verified by the leverage approach, which is based in computing the leverage, h*, for each compound for which the QSPR model is used to predict the property under study. The warning leverage is generally fixed at 3k/n, k being the number of model parameters and n being the number of training set compounds (Fig. 4).

## Artificial neural network

Artificial neural networks (ANNs) [31-33] are biologically inspired algorithms designed to simulate the way in which the human brain processes information. ANNs are parallel computational models which are able, at least in principle, to map any nonlinear functional relationship between an input and an output hyperspace to the desired accuracy. The network receives a set of inputs, which are multiplied by each neuron’s weight. These products are summed for each neuron, and a nonlinear transfer function is applied. A set of six descriptors that were appeared in the MLR model, were used as input parameter of the network.

In this investigation, the *logsig function* was used as a transfer function of hidden layer, *traingdx function* as a training function and a linear function for the output layer. We optimized the parameters such as number of nodes, momentum (*α*) and learning rate (*η*). These values were obtained during the network training. The initial weights were randomly selected between (-0.3 and 0.3). A neural network with 6-6-1 topology was developed with optimum momentum and learning rate.

Stepwise MLR is a simple and powerful method to modeling the linear correlation of significant input variables but it does not account for nonlinear relationship, so ANN model was constructed for this purpose. To compare the performance of the MLR and ANN models descriptors that used in the MLR model should be the same as the input variables for generation the network. The parameters and results of these models are shown in Table 4. As it is obvious from the results in Table 4 ANN has more or less same efficiency as well as MLR to map the relationship between input objects and response values.

**Table 4:** Parameters of the ANN model

optimized parameters of the ANN				
Transfer function				Logsig
Training function				Traingdx
Number of input neurons				6
Number of hidden neurons				6
Number of output neurons				1
Learning rate				0.5
Momentum				0.5

**R^2^**	**R^2^_cal_**	**R^2^_pre_**	**RMSEC ^a^**	**RMSEP ^b^**

0.977	0.983	0.967	0.173	0.315

^a^ Root mean squared error of calibration

^b^ Root mean squared error of prediction

## Description of models descriptors

Molecular descriptors will probably play an increasing role in scientific growth. In fact, the availability of large numbers of theoretical descriptors containing diverse source of chemical information would be useful to better understand relationship between molecular structure and experimental evidence.

The X1sol (solvation connectivity index) and X5A(average connectivity index) appearing in the MLR model mainly show the topological characteristics. These indicate that dispersion interactions and the extent of branching of the molecules affect the retention behavior of VOCs on the column. Solvation connectivity indices , eq. (3), defined as:(3)

where L*_a_* is the principal quantum number (2 for C, N, O atoms,3 for Si, S, Cl, …)of the *a* th atom in the *k* th subgraph and δ_a_ the corresponding vertex degree; k is the total number of *m* th order subgrah; *n* is the number of vertices in subgraph. The normalization factor 1**/** (2*^m^*^+^*^1^*) is defined in such a way that the indices *^m^*χ and *^m^*χ*^s^* for compounds containing only second-row atom coincide. The X1sol shows a regression coefficient (0.823), which is the largest among the descriptors appearing in the model. This parameter can be considered as entropy of solvation and somehow indicates the dispersion interactions occurring in the solutions. X5A also is a measure of branching of the molecules. The large contributions of this parameter in the retention behavior of VOCs are in agreement with the contribution that one would expect for the interaction of a stationary phase with the VOCs. X5A is the average connectivity index that can be obtained from molecular graph.

MATS2m (Moran autocorrelation− lag 2/weighted by atomic masses) and ATS2m (broto-moreau autocorrelation of a topological structure- log2/weighted by atomic masses) are 2D autocorrelation descriptors [34], which are also obtained from molecular graphs, by summing the products of atom weights of the terminal atoms of all the paths of the considered path length (the lag). The MATS2m and ATS2m are related to the atomic property of a molecule, these descriptors concerning molecular size, which influence retention of compound. The term ATS2m is a graph invariant describing now the considered property (retention time) is distributed along the topological structure. Molecular descriptors based on the autocorrelation function AC*_l_* defined as:

where *f*(*x*) is any function of the variable *x* and *l* is the lag representing an interval of *x*; *a* and *b* defined the total studied interval of the function. The function *f*(*x*) is usually a time dependent function such s a time – dependent electrical signal, or spatial dependent function such as the population density in space.

Mor21m (3D MoRSE- signal 21/weighted by atomic masses) and Mor10m (3D MoRSE- signal 10/weighted by atomic masses) are among the 3D-MoRSE descriptors. 3D-MoRSE [35] descriptors (Mor21m, Mor10m) are molecule atom projections along different angles, such as in electron diffraction. They represent different views of the whole molecule structure, although their meaning remains not too clear. 3D-MoRSE descriptors are based on the idea of obtaining information from the 3D atomic coordinates by the transform used in electron diffraction studies for preparing theoretical scattering curves. 3D-MoRSE descriptors are important because they take into account the 3D arrangement of the atoms without ambiguities (in contrast with those coming from chemical graphs), and also because they do not depend on the molecular size, thus being applicable to a large number of molecules with great structural variance and being a characteristic common to all of them.

All of these descriptors are individually important to increase or decrease the retention times but the final effect is not result in each descriptor individually and all of the selected descriptors as a group can influenced on the dependent variable. This problem in many cases led to the hard interpretation of the model.

## Conclusion

QSRR analysis was performed on a series of volatile organic compounds, for prediction the retention times. Stepwise regression was used to as variable selection method. The comparison of the models that were developed by selected descriptors shows that MLR can present the satisfactory result for predicting of retention time of VOCs. Comparison of the linear (MLR) and nonlinear (ANN) methods showed the little superiority of the ANN over the MLR model for the prediction of the retention time of VOCs, so the results suggest that a relation between the molecular descriptor and the retention times is linear. The QSRR models proposed with the simply calculated molecular descriptors can be used to estimate the chromatographic retention times of new compound even in the absence of the standard candidates. The most important selected descriptors are topological which can capture the variance in the retention times what related to the size and shape of the molecules.

## Competing interests

The authors declare that they have no competing interests.
